# The impact of teaching psychological welfare on marital satisfaction and self-efficacy in nurses

**Published:** 2015

**Authors:** B Sabzianpoor, M Ghazanfari Amrai, M Jalali Farahani, R Soheila, A Mahdavi, S Rahmani

**Affiliations:** * Psychometric Psychology, Islamic Azad University, Central Tehran Branch, Iran; ** General Psychology, Islamic Azad University, Science and Research Branch, Semnan, Iran; *** General Psychology, Tarbiat Modares University, Tehran, Iran; **** Clinical Psychology, Islamic Azad University, Chaloos Branch, Iran; ***** Department of Psychology, Faculty of Psychology and Educational Sciences, University of Payame Noor, Tehran, Iran; ****** Student Health Psychology, Islamic Azad University, Karaj Branch, Iran

**Keywords:** psychological welfare, marital satisfaction, self-efficacy, nurses

## Abstract

**Objective:** Proper psychological interventions for enhancing public health and mental welfare in nurses are of great importance. This study intended to explore the influence of the teaching psychological welfare on marital satisfaction and self-efficacy in nurses of Imam Khomeini hospital.

**Methodology:** The method of the present study was semi-experimental with a pre-test post-test design and a control group. Hence, 24 nurses were selected from Imam Khomeini hospital, by using convenience sampling, and they were divided into two groups: experiment and monitoring. By using a 47-questions marital satisfaction questionnaire and a 17-questions general self-efficacy questionnaire, both groups were pre-tested. Then the test group, received lessons on psychological welfare within ten sessions, but the control group received no interventions. Afterwards, both groups were post-tested, and the data collected were analyzed by using descriptive and inferential statistics methods through SPSS software.

**Findings:** Findings showed that teaching psychological welfare significantly increased marital satisfaction and self-efficacy in nurses (p < 0.001).

**Conclusion:** From this research, it was concluded that teaching psychological welfare is an effective strategy for those with risky jobs like nurses, because it is highly efficient, especially when it is performed in groups, because it is cheap, and because it is acceptable by different working people.

## Introduction

Human forces are the most valuable assets in every organization; the higher the quality of these assets, the more productive and resistant the organizations will be [**1**]. One of the most robust systems in every society is hospitals, because they are directly connected to the individuals’ health. Nurses form the biggest part of the hospital staff. Therefore, recruiting and maintaining them is vital [**2**]. Nurses are individuals who are responsible for controlling and monitoring hospitals 24 hours a day; hence, they are continually exposed to numerous stressors [**3**]. Many examinations showed that 93 percent of the nurses are regularly affected by stressors in work environments, which can also affect their physical and mental health [**4**]. Therefore, paying attention to the factors that improve the nurses’ mental status is of great importance.

It is an undeniable fact that for every individual, work life and family life are two crucial fields. In spite of the fact that they are seemingly two separate areas in the humans’ life, the cause and effect dynamisms of families and work have been emphasized by scientists since years ago [**5**]. Every person’s satisfaction with either field significantly affects different aspects of life. Regarding family factors, marital satisfaction is one of the most important factors, which lead to a healthy family performance; playing a crucial role in the endurance of marriage lives. Also, it can have a big impact on other aspects of life such as jobs [**6**]. Marital satisfaction is an index of healthy marital relationships, including the individuals’ emotional, cognitive, and intellectual perceptions of their marriage life. Andreassen CS, Hetland J, Stale Pallesen S (2013) [**5**] believe that marital satisfaction is the adjustability of the status quo and the expected status. Based on this definition, marital satisfaction happens when the status quo matches what couples expect in their marital relationships. Also, in literature related to marital satisfaction, the presented definition was ignored by Ellis. Andreassen CS, Hetland J, Stale Pallesen S (2013) [**6**] define marital satisfaction as something which includes feelings of joy and being pleased with all relationships. In other words, marital satisfaction is the couples’ general assessment of marital relationships. This general assessment could be a reflection of the individuals’ happiness with the marital life or a combination of their satisfaction with the aspects related to marital relationships [**7**]. Therefore, by emphasizing different positive aspects, marital satisfaction could significantly affect the individuals’ lives; hence, it requires attention and examinations.

In addition to the marital satisfaction, another variable which is imperative in the individuals’ professional and even personal lives, and which should be examined more carefully, is the feeling of being self-efficient, especially in nurses. Self-efficacy is an assuredness that an individual has successfully performed a particular behavior and waits for the expected results. In fact, it is a person’s belief in achieving a particular goal [**8**]. As a factor which significantly influences resources to achieve goals (Vancouver, More and Yoder, 2008), self-efficacy is one of the valuable items that receives a lot of attention from positive psychology [**9**]. It was conceptualized as a belief in the organization and doing necessary activities for achieving a set goal. In addition, using a social-cognitive approach to jobs, self-efficacy helps form positions and motivations directly and indirectly (by affecting the expected results) [**10**]. High self-efficacy regulates the level of stress; high self-esteem increases welfare and physical health, and it helps recover quickly from chronicle diseases [**11**]. Also, a low self-efficacy leads to anxiety, depression, and low intellectual welfare [**12**]. Therefore, the examining factors that enhance self-efficacy are crucial for clinical researchers. 

One of the most important psychological interventions for the improvement of psychological conditions and mental health is teaching psychological welfare. Psychological welfare is defined as the development of the individuals’ real talents, including six components: purpose in life, positive relations with the others, personal growth, self-acceptance, autonomy, and environmental mastery [**13**]. In various investigations, it was noted that with a change in psychological welfare, it is possible to predict the individuals’ perceived eligibility and health [**14**]. Hence, the present study was conducted to examine the effect of teaching psychological welfare on marital satisfaction and self-efficacy in nurses of Tehran’s Imam Khomeini Hospital. 

## Methodology

The current research was semi-experimental with a pre-test post-test design and a control group. The statistical community of the study consisted of all the nurses working in Tehran’s Imam Khomeini Hospital (autumn, 2016), who were selected by using the convenience sampling method. To calculate the sample size, 12 people were chosen for each group, considering the fact that the minimum sample size for the experiment samples was 12 [**15**]. To increase the statistical competence and manage probable drops in the number of participants, the sample size was 24 (n = 24) for each group. Participants were selected from all the nurses working in Tehran’s Imam Khomeini Hospital, by using a voluntary non-random sampling method. Criteria for entering the present study included conscious tendency to participate, ability to take part in sessions and doing assignments, cooperating to complete the tools, having an age range of 25 to 45, having a minimum education (diploma), having a psychological balance, and having a physical balance. The criteria for exiting the study were the following: not having a tendency to participate, being absent for more than three sessions, not having the ability to take part in sessions and do assignments, and having a history of training or psychological therapy, which was not part of the present study. 

The method of performing the research included the selection of some nurses working in Tehran’s Imam Khomeini Hospital, by using a voluntary non-random sampling method; and when they had the entrance criteria, they were randomly divided into two groups: experiment and control. Before conducting the study, the ethical principles were observed. First, participants were given precise explanations on the purpose of the research and the effect of such studies on the psychological conditions; then they were asked whether they wanted to participate in the study or not. Afterwards, the researcher collected all the information connected to the participants, and they were assured that these data were confidential and kept by the researcher. Next, the experiment group received group lessons on psychological welfare within ten sessions, but the control group received no interventions. Finally, both groups were post-tested. The protocol of psychological welfare training sessions is presented in **Table 1**.

The tools used in the present study included a demography questionnaire, Sherer’s et al. general self-efficacy questionnaire, and Enrich’s marital satisfaction questionnaire. 

Demography questionnaire: The demographic questionnaire included items such as age, gender, education, field of study, and marital status. This survey was provided and evaluated by the researchers of this study.

Enrich’s short-form marital satisfaction questionnaire: This questionnaire was designed by Olson et al. [**16**] to evaluate the potential problematic contexts and identify the settings for power and marital relationships. This test had two forms, one with 115 questions, and the other with 125 questions, which were composed of 12 sub-tests. Because the primary form had multiple questions, it tired respondents. In addition, they were provided a short form of this questionnaire that had 47 questions. Using Cronbach’s alpha coefficient, the reliability of the primary form of Enrich’s marital satisfaction survey was calculated to be 0.92. Using Cronbach’s alpha coefficient, Soleimanian calculated the reliability of the short-form questionnaire to be 0.90 [**15**]. In the present study, the short form of the questionnaire was used [**12**]. Amiri Majd M, Zari Moghadam F (2011) [**16**] did an extensive research on the validity of the test. By using the retest, they calculated the reliability of the trial to be between 0.65 and 0.94 and reported its difference to be 0.90. Examinations were conducted with the help of this scale, for happy, consistent, and divorced couples and those who wanted to get divorced approved the validity of this magnitude. 

General self-efficacy questionnaire (G.S.E.S): this scale made by Sherer et al. (1982) included 17 items. The Self-efficacy Questionnaire measures the individuals’ capability of coping with different situations. This questionnaire was set based on a five-point Likert scale; “I completely disagree” responses received a score of 1, and “I completely agree” responses received a score of 5; high scores showed a high self-efficacy in people. When marking this questionnaire, questions 3, 8, 9, 13, and 15 were in line with “agreement”, and the rest of the questions were marked in a reverse format. The reliability and validity of the questionnaire: To analyze the data, “SPSS-20” software was used. To analyze the research data in a descriptive statistical level, the statistical method used indexes such as average, standard deviation, rate, and frequency percentage; and in an inferential statistical level, it used the tests of a single-variable multivariate covariance analysis model.

**Table 1:** Protocol of psychological welfare training sessions

session	Subject
first	Teaching and introducing psychological welfare, and explaining why mental welfare came into existence? And questioning individuals what brings happiness.
second	Teaching how to accept oneself (how to accept ourselves with negative and positive features, how to accept what we are, how to put up with our past conduct and like ourselves).
third	Second part of last session’s discussion: Teaching self-knowledge (how to get to know ourselves, how to have a correct attitude to ourselves, and how to become familiar with our personality traits).
fourth	Teaching how to positively communicate with others (introducing the concept of relationship and its types, becoming familiar with communicational skills and methods of an effective communication).
fifth	Second part of last session’s discussion: Teaching optimism and positive thinking, and that optimism and positive thinking play a role in positive communications with the others.
sixth	Third part of the fourth session’s discussion: Teaching the essence of marital satisfaction and self-efficacy, how to enhance them, and why people with these features are more successful.
seventh	Teaching independence and autonomy (teaching lessons on how to trust others and oneself, teaching assertiveness, how to say “no”, and reinforcing these skills in respondents).
eighth	Teaching and introducing the concept of personal growth and that experiencing new things increases personal growth.
ninth	Teaching how to dominate the environment (how to manage life and how to manage circumstances and the environment, and explanations on the advantages of managing time and correct planning).
tenth	Teaching how to set goals for life (the ability to find meaning, purpose, and orientation in life, explaining the advantages of being purposeful and setting goals with prioritization).

**Research Findings**

The demographic characteristics of the sample in the present study are presented in **Table 2**.

**Table 2:** Demographic characteristics of respondents

variable	group	frequency	Frequency percentage	Mean and standard deviation
Age	25 to 30 years	8	20	
	31 to 35 years	12	30	
	36 to 40 years	11	27/5	
	41 to 45 years	9	22/5	
education	BA	22	55	
	MA and upper	18	45	

As shown in **Table 1**, the most frequency of the respondents in the present study was related to individuals between the ages of 31 and 35, with 12 members (30 percent). Moreover, the least frequency of the respondents was related to people between the ages of 25 and 30, with eight members (20 percent). Also, the average of those “surveyed'” years was 34/ 15, and the standard deviation was 7/ 36. The other data related to the demographic features of the present study are presented in **Table 2**.

**Table 3:** Descriptive statistics of marital satisfaction and self-efficacy scores in both groups, with a pre-test post-test design

component	index	experiment		control	
		Pre-test	Post-test	Pre-test	Post-test
Marital satisfaction	Mean	138.80	165.05	138.40	139.41
	Standard deviation	110.04	13.14	10.57	9.82
Self-efficacy	Mean	36.15	48.01	40.90	41.40
	Standard deviation	5.01	8.11	5.75	6.05

As shown in **Table 3**, in the experiment group, the mean of the marital satisfaction and self-efficacy increased in the post-test stage, compared to the control group. 

**Table 4:** Results of Loin’s test for the examination of the consistency of variances of marital satisfaction and self-efficacy in the post-test stage

variable	stage	F	Degree of freedom 1	Degree of freedom 2	Significance level
Marital satisfaction	Post-test	0.862	1	38	0.359
Self-efficacy	Post-test	0.461	1	38	0.502

As shown in **Table 4**, the hypothesis of zero about the equality of both groups’ variances in marital satisfaction and self-esteem was approved. It means that for marital satisfaction and self-efficacy, the variances of both groups were equal in the society, with no significant differences. Therefore, following Loin’s pre-hypothesis, it was possible to perform a covariance analysis of the results to examine the hypothesis of the study. 

**Table 5:** The results of the multivariate covariance analysis for the post-test scores with the pre-test control in marital satisfaction and self-efficacy

Test title	value	F	Degree of freedom	Significance level	Eta square	competence
Pylayy effect	0.593	26.921	2	0.001	0.593	0.95
Wilks Lambda	0.407	26.921	2	0.001	0.593	0.95
Hotelling effect	1.455	26.921	2	0.001	0.593	0.95
Ray’s largest root	1.455	26.921	2	0.001	0.593	0.95

As presented in **Table 5**, a significance level of all tests (p > 0.001) showed that at least in one of the dependent variables (marital satisfaction and self-efficacy), there was a significant difference between the two groups. Moreover, based on the eta square, 0.59 percent of the differences between the individuals were related to the effect of independent variable, i.e. intervention method (teaching psychological welfare). On the other hand, because the statistical competence was 0.95, which was higher than 0.80, the sample size for performing the research was acceptable. The results were connected to the significant difference of each dependent variable presented below.

**Table 6:** The results of the multivariate covariance analysis for the examination of the effect of teaching psychological welfare on marital satisfaction and self-efficacy in the post-test stage

index	Sum of squares	Degree of freedom	Mean of squares	F	Significance level	Eta square
Marital satisfaction	435.601	1	435.601	8.494	0.005	0.183
Self-efficacy	6477.025	1	6477.025	48.112	0.001	0.559

Based on the data presented in **Table 6**, since the significance level was p > 0.001, the difference between the marital satisfaction and the self-efficacy between both groups was approved. Also, it was stated that 0.18 percent of the changes in the score of marital satisfaction and 0.55 percent of the variations in the score of self-efficacy were due to the independent variable (teaching psychological welfare). Therefore, it could be said that teaching psychological welfare increases marital satisfaction and self-efficacy in nurses. 

## Discussion and Conclusion

Regarding the fact that the current study was aimed at examining the effect of teaching psychological welfare on the marital satisfaction and self-efficacy in nurses of Imam Khomeini Hospital, findings obtained from single-variable and multivariate covariance analysis showed that teaching psychological welfare significantly increased the marital satisfaction and self-efficacy in nurses of Imam Khomeini Hospital. It was in congruence with the other findings obtained from studies conducted by Parvin N, Fatemi A, Aminian F, Rafiee L (2015), Pourghaffari S, Pasha GHR, Attari YA (2010), Shakarami M, Davarnia R, Zahrakar K, Gohari S (2015) [**17**-**19**].

To express their similar findings, Parvin N, Fatemi A, Aminian F, Rafiee L (2015) [**17**] stated that the psychological lessons on marriage enabled individuals to exchange their messages explicitly. Also, couples without such skills could continue their marriage life. On the other hand, not using life competencies in a correct way might lead to disorders in life as well as collapses in relationships. To express their similar findings, Pourghaffari S, Pasha GHR, Attari YA (2010) [**18**] stated that those nurses who manage to implement strategies in their lifestyles after they finish training sessions will be able to make differences in their marriage lives. If they have complaints about their relationships with their husbands and if they feel that they cannot adaptively act in situations, they will not feel that their needs and intentions are fulfilled. Thus, they will not be pleased with their marriage lives. However, changes in life patterns and producing interest in participants of psychological welfare training sessions led to a new attitude to marital relationships followed by positive emotions and hopefulness. Individuals who manage to make a difference in their lifestyles, after finishing training sessions, come to a new philosophy of life and will be able to enjoy their lives. 

The finding that teaching psychological welfare increases self-efficacy in nurses is in congruence with the findings obtained from studies conducted by Fallahian R, Aghaie A, Atashpoor A, Kazemi A (2014), Asghari F, Saadat Atefi Korjundani S, Janalizadeh Kokneh S (2015), Peymani N, Ezzati Rastegar Kh (2013), Jannati Jahromi M, Moein L, Yazdani L (2011), Soltani Majd AH, Taghizadeh ME, and Zareh (2015) [**14**,**20**-**23**]. To express their similar findings, Fallahian R, Aghaie A, Atashpoor A, Kazemi A (2014) [**14**] stated that teaching psychological welfare has numerous advantages, the most important of which are an improvement in inter-personal relationships, self-acceptance, dominance over the environment and so on, which lead to an increase in the self-esteem and self-efficacy of individuals. Also, improvements in the mental states of people after participating in the training sessions can be referred to, which are important factors helping in the improvement of self-efficacy. Self-efficacy is the product of the past conditions and failures. If by training, individuals are offered solutions to coming to terms with the failures leading to success, it could be hoped that they will resort to challenging assignments and handling them. Therefore, teaching psychological welfare increases self-efficacy.

**Acknowledgements**

The authors would like to thank the officials of Imam Khomeini Hospital for their help. The authors would also like to thank all the nurses who participated in this research. 
